# Quality improvement in managing patients with non-muscle-invasive bladder cancer by introducing a surgical checklist for transurethral resection of bladder tumor

**DOI:** 10.1371/journal.pone.0276816

**Published:** 2022-10-27

**Authors:** Hiroshi Kikuchi, Takahiro Osawa, Takashige Abe, Ryuji Matsumoto, Satoru Maruyama, Sachiyo Murai, Nobuo Shinohara

**Affiliations:** 1 Department of Urology, Hokkaido University Graduate School of Medicine, Sapporo, Japan; 2 Department of Urology, Teine Keijinkai Hospital, Sapporo, Japan; 3 Department of Urology, National Hospital Organisation Hokkaido Cancer Center, Sapporo, Japan; Osaka Medical Center for Cancer and Cardiovascular Diseases, JAPAN

## Abstract

**Background:**

The quality of transurethral resection of bladder tumor (TURBT) markedly varies among surgeons and may have a considerable impact on treatment outcomes. The importance of a surgical checklist for TURBT has been suggested in order to standardize the procedure and improve surgical and oncological outcomes. In the present study, we verified the usefulness of a checklist for managing patients with non-muscle-invasive bladder cancer (NMIBC).

**Methods:**

This retrospective study included 201 NMIBC patients diagnosed with Ta, T1, or Tis between October 2011 and February 2021. After September 2016, TURBT was performed with a checklist. We analyzed the intravesical recurrence-free survival (RFS) rate and the presence or absence of the detrusor muscle in resected specimens before and after the introduction of the checklist. Survival rates were compared using the Log-rank test. A multivariate analysis with Cox proportional hazards modeling was performed to verify risk factors for intravesical recurrence.

**Results:**

Ninety-nine patients who underwent TURBT with the checklist (checklist group) were compared with 102 patients who underwent TURBT without the checklist (non-checklist group). When the analysis was narrowed down to 9 critical items, we observed a mean number of 9 documented items per operative report (98.0% completion) after implementation of the checklist. Two-year intravesical RFS rates in the checklist and non-checklist groups were 76.7 and 69.5%, respectively (p = 0.1059). The Cox proportional multivariate analysis showed that the rate of intravesical recurrence was slightly lower in the checklist group (hazard ratio 0.7376, 95% CI 0.4064–1.3388, P = 0.3170).

**Conclusion:**

The introduction of a checklist is recommended for the standardization of TURBT and increasing the quality of operative reporting, and it may also improve oncological outcomes.

## Introduction

Non-muscle-invasive bladder cancer (NMIBC) accounts for approximately 75% of initially diagnosed bladder cancers [1]. Transurethral resection of bladder tumor (TURBT) is required as an initial intervention for bladder cancer and is one of the most common endoscopic urologic procedures. The objectives of initial TURBT are to confirm all macroscopic diseases, establish the tumor grade with accurate pathological staging, and identify clinically important prognostic factors [2]. The quality of TURBT markedly varies among surgeons and institutions, which may have a considerable impact on treatment outcomes [3]. Pan and Soloway initially suggested the importance of a surgical checklist for TURBT to standardize the procedure and improve surgical and oncological outcomes [4]. Anderson et al. reported that a 10-item checklist improved the reporting of crucial procedural elements [5]. Suarez-Ibarrola et al. showed that an 8-item surgical checklist reduced the recurrence rate in patients with NMIBC and was independently associated with significant improvements in recurrence-free survival (RFS) [6]. Taoka et al. also demonstrated that surgical checklists during TURBT in clinical practice increased the quality of procedures and reduced the recurrence rate in patients with NMIBC [7].

We introduced a modified version of Anderson’s checklist for TURBT. In the present study, we verified the usefulness of the checklist for managing patients with NMIBC. We retrospectively analyzed the intravesical RFS rate and presence or absence of the detrusor muscle in resected specimens before and after the introduction of the checklist.

## Materials and methods

### Study population

Between October 2011 and February 2021, 274 patients underwent TURBT at Hokkaido University Hospital. A total of 201 patients who had undergone TURBT and were pathologically diagnosed with Ta, T1, or Tis bladder cancer were selected. Institutional Review Board approval was acquired from Hokkaido University Hospital (017–0162). Patient characteristics, including age, sex, the tumor characteristics, pathological outcomes, and perioperative complications, were retrospectively collected from our database and medical charts.

### Treatment and follow-up protocol

TURBT was always performed by a resident under the supervision of one of the senior staff and other residents. After September 2016, TURBT was performed according to a modified version of Anderson’s checklist (S1 Table). This checklist consists of 9 critical items that need to be recorded and performed as the minimum during every high-quality TURBT and the other three items about the purpose of TURBT and the biopsy site. These items include the visual appearance of tumors and descriptions of the tumor size and number. We evaluated the quality of the TURBT operative report by retrospectively counting the number of item elements in each operative report for TURBT. Patients with high-grade Ta/T1 tumors were indicated for second TUR and the instillation of bacillus Calmette-Guérin (BCG), which were sometimes omitted at the discretion of the attending doctor based on the status of the patient (elderly or with comorbidities). After the induction of BCG or last TURBT, cystoscopy and urine cytology were performed every 3 months during the first 2 years and every 6 months thereafter.

### Statistical analysis

Survival rates were compared using the Log-rank test. Patient characteristics, the rate of the presence of the detrusor muscle, and perioperative complications were compared using the chi-squared test or ANOVA. A multivariate analysis with Cox proportional hazards modeling was performed to identify independent predictive factors for intravesical recurrence. The time to recurrence was defined as the time from the date of initial TUR to that of first intravesical recurrence. Intravesical recurrence was defined as any type of recurrence in the bladder. Disease progression was defined as development of muscle invasive disease or metastatic disease. The follow‐up of patients without the recurrence of bladder tumors was censored to the date of their last follow-up. Patients who underwent cystectomy were censored at the date of cystectomy. JMP version 16 (SAS Institute, Japan) was used for all analysis, and p<0.05 was considered to be significant.

## Results

The clinical and pathological features of the 201 patients examined are shown in Table 1. One hundred and two patients underwent TURBT without the checklist; their median age was 70 years (interquartile range, 65–76) and 68.6% were male. The pathological tumor grade at first TUR was low in 47 (46.1%) cases and high in 53 (52.0) cases. Pathological T staging was pTa in 73 (71.6%) cases, pT1 in 26 (25.5%), and primary CIS in 3 (2.9%). Among Ta/T1 cases, concurrent CIS was detected in 8 (8.1%) patients. Ninety-nine patients underwent TURBT with the checklist; their median age was 73 years (interquartile range, 67–80) and 77.8% were male. The pathological tumor grade at first TUR was low in 30 (30.3%) cases and high in 69 (69.7) cases. Pathological T staging was pTa in 63 (63.6%) cases, pT1 in 28 (28.3%), and primary CIS in 8 (8.1%). Among Ta/T1 cases, concurrent CIS was detected in 12 (13.2%) patients. The proportion of high-grade tumors and the patients who underwent BCG therapy were significantly higher in patients who underwent TURBT with the checklist (P = 0.0012 and P = 0.0125). No significant differences were observed in other factors between the two groups.

**Table 1:** Distribution of patient characteristics.

Variables	Checklist		p-value
	No	Yes	
	n = 102	n = 99	
**Preoperative factors**			
Age, year, median (interquartile range)	70 (65–76)	73 (67–80)	0.0602
Sex, no. (%)			
Male	70 (68.6)	77 (77.8)	0.1434
Female	32 (31.4)	22 (22.2)	
Tumor status			0.4655
Primary	86 (84.3)	87 (87.9)	
Recurrent	16 (15.7)	12 (12.1)	
Tumor multiplicity, no. (%)			0.6829
Single	42 (41.2)	44 (44.4)	
Multiple	59 (57.8)	55 (55.6)	
Unknown	1 (1.0)	0 (0.0)	
Tumor size, no. (%)			0.2843
<3 cm	87 (85.3)	79 (79.8)	
≥3 cm	13 (12.7)	18 (18.2)	
Unknown	2 (2.0)	2 (2.0)	
**Postoperative factors**			
Histology, no. (%)			0.6741
UC	95 (93.1)	95 (96.0)	
UC with glandular differentiation	5 (4.9)	3 (3.0)	
UC with squamous differentiation	2 (2.0)	1 (1.0)	
Pathological tumor grade, no. (%)			0.0012
Low	53 (52.0)	30 (30.3)	
High	47 (46.1)	69 (69.7)	
Unknown	2 (1.9)	0 (0.0)	
pT stage, no. (%)			0.2189
pTa	73 (71.6)	63 (63.6)	
pT1	26 (25.5)	28 (28.3)	
Primary CIS	3 (2.9)	8 (8.1)	
Concurrent CIS*, no. (%)	8 (8.1)	12 (13.2)	0.2519
Second TUR, no. (%)			0.0567
Yes	33 (32.4)	45 (45.5)	
No	69 (67.6)	54 (54.5)	
Residual tumor at second TUR, no. (%)			0.8848
Yes	13 (39.4)	17 (37.8)	
No	20 (60.6)	28 (62.2)	
Adjuvant intravesical therapy			0.0125
BCG	28 (27.5%)	47 (47.5%)	
Chemotherapy	4 (3.9%)	2 (2.0%)	
No	70 (68.6%)	50 (50.5%)	

*Concurrent CIS rates were counted in Ta/T1 cases.

BCG = bacillus Calmette-Guérin; CIS = carcinoma in situ; TUR = transurethral resection; UC = urothelial carcinoma.

**Fig 1:**
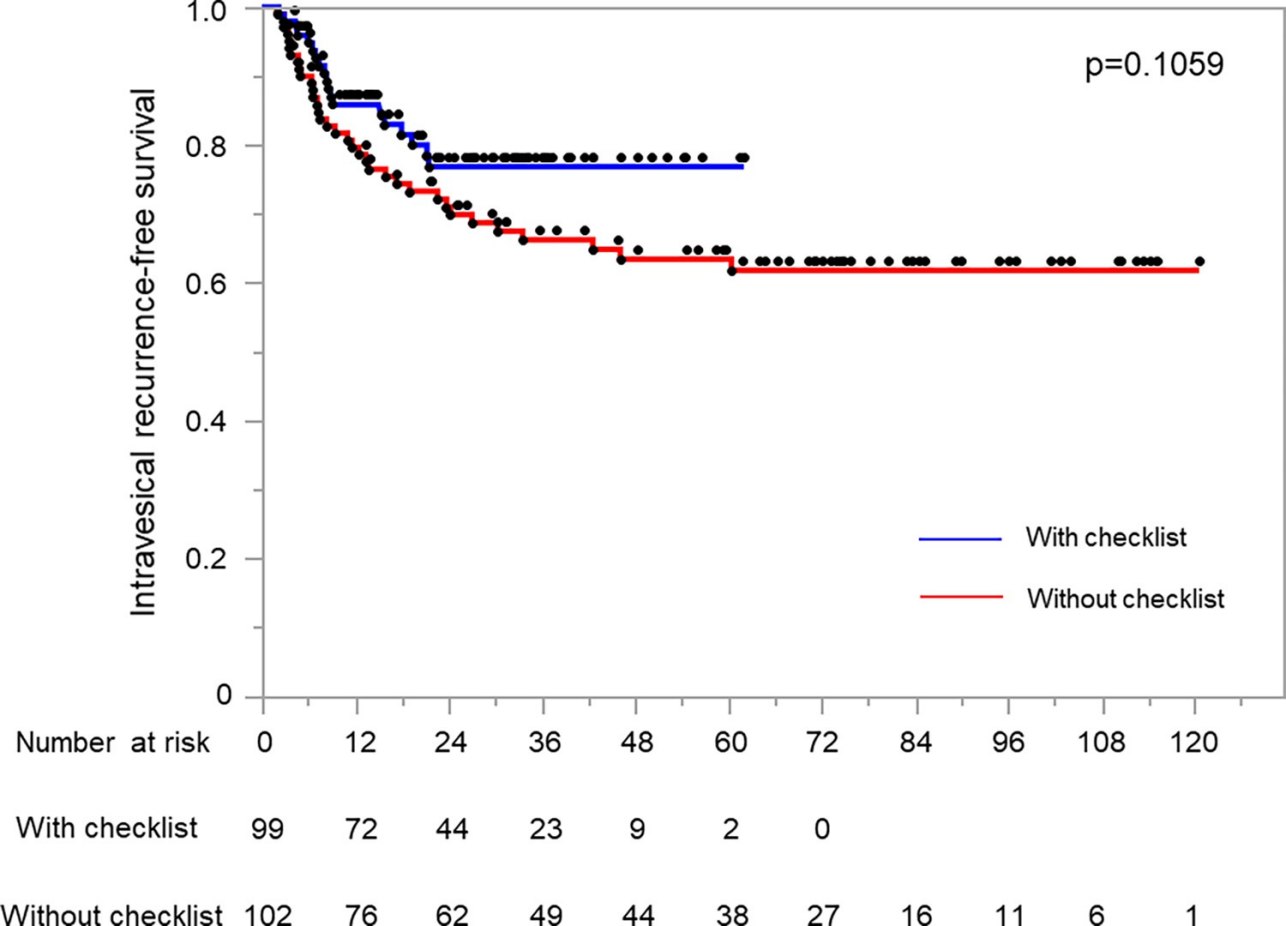
Kaplan–Meier curves of the intravesical recurrence-free survival rates for TURBT using with and without surgical checklist groups.

When the analysis was narrowed down to 9 critical items, the mean number of critical procedural elements included in the TURBT operative report was 9 per operative report after the implementation of the checklist (98.0% completion). Two-year intravesical RFS rates in the checklist and non-checklist groups were 76.7% (median follow-up: 28.6 months) and 69.5% (median follow-up: 70.7 months), respectively (p = 0.1059, Fig 1). In the patients received adjuvant intravesical therapy, the intravesical RFS rates were almost same between the group with and without checklist (p = 0.9950, S1A Fig). On the other hand, the intravesical RFS rate of non-checklist group tended to decrease compared to that of checklist group in the patients not received adjuvant intravesical therapy (p = 0.1315, S1B Fig). There was not a significant difference in the presence or absence of the checklist in the group with and without adjunctive intravesical therapy. Table 2 shows the results of the multivariate analysis including factors that influenced intravesical recurrence and the use of the checklist. The hazard ratio slightly decreased in the checklist group (hazard ratio 0.8013, 95% CI 0.4229–1.5184, P = 0.4969). There were no factors that influenced disease progression (S2 Table). Table 3 shows the perioperative outcomes. After the introduction of the checklist, no significant changes were noted in the presence of the detrusor muscle (non-checklist group: 90.9% vs. checklist group: 90.2%, p = 0.8629). In addition, no significant differences were observed in the incidence of perioperative complications requiring reoperation or readmission before or after the introduction of the checklist (non-checklist group: 5.9% vs. checklist group: 4.0%, p = 0.5482).

**Table 2:** Multivariate analysis with Cox proportional hazards modeling for intravesical recurrence.

Variables	N	Multivariate	
		Hazard ratio (95% CI)	*P*-value
Tumor status			
primary	173	Ref	
recurrent	28	1.6410 (0.8274–3.2546)	0.1563
Number of tumors			
single	86	Ref	
multiple	114	3.3905 (1.7042–6.7452)	0.0005
Tumor size			
<3 cm	167	Ref	
≥3 cm	31	1.5908 (0.7085–3.5716)	0.2606
Histology			
UC	190	Ref	
UC with variant histology	11	1.5729 (0.5671–4.3626)	0.3842
Pathological tumor grade			
Low	83	Ref	
High	116	0.8849 (0.3411–2.2956)	0.8015
TUR with checklist			
no	102	Ref	
yes	99	0.8013 (0.4229–1.5184)	0.4969
Second TUR			
no	120	Ref	
yes	81	1.3004 (0.5252–3.2199)	0.5702
Adjuant therapy (BCG or chemotherapy)			
no	120	Ref	
yes	81	0.3111 (0.1430–0.6768)	0.0032

BCG = bacillus Calmette-Guérin; CIS = carcinoma in situ; TUR = transurethral resection; UC = urothelial carcinoma.

**Table 3:** Perioperative outcomes.

Variables	Checklist		p-value
	No	Yes	
	n = 102	n = 99	
Operation time, min, median (interquartile range)	46 (31–65)	52 (32–76)	0.0386
Presence of the detrusor muscle, no. (%)	92 (90.2)	90 (90.9)	0.8629
Perioperative complications*, no. (%)	6 (5.9)	4 (4.0)	0.5482
Length of postoperative hospital stay, day, median (interquartile range)	9 (7–11)	8 (7–9)	0.0761
Urinary catheter duration, day, median (range)	3 (1–12)	3 (1–13)	0.3979

*Requiring reoperation or readmission

## Discussion

Since the implementation of the checklist developed by the World Health Organization as the standard of care in current clinical practices worldwide, the outcomes of various types of surgery have been improved [8]. TURBT-specific surgical checklists were recently shown to be therapeutically beneficial in bladder cancer surgery [6, 7]. The present results also showed that the intravesical RFS rate slightly decreased after the introduction of the checklist despite the high proportion of high-grade tumors in the checklist group.

In contrast to the present study, previous findings showed a significant improvement in RFS before and after the introduction of the checklist [6, 7]. One of the reasons why the present study did not show any significant difference in RFS before and after the introduction of the checklist was that the RFS rate was high even before the introduction of the checklist; therefore, any improvement was small. De Jager et al. also reported that a surgical checklist was less effective at decreasing complications in hospitals with a low rate of complications [8, 9]. However, further decreases in the rate of recurrence are expected, even in patient populations with a low recurrence rate at baseline.

In the present study, the RFS rate at 2 years was as high as 70% prior to the introduction of the checklist. One of the reasons for the lower RFS rate than in previous studies (less than 50%) was that the proportion of TURBT with a muscular layer (more than 90%) in the first transurethral resection specimen was high [6, 7]. Mariappan et al. previously reported that a surgeon’s experience affected the detrusor muscle collection rate as well as the intravesical recurrence rate [3]. In our hospital, TURBT was carefully and meticulously performed and approved by several on-site doctors, which increased the muscle layer collection rate and improved treatment outcomes.

We observed a significant increase in the number of reported checklist items after its implementation in the present study. Although it currently remains unclear whether more accurate reporting was associated with a lower tumor recurrence rate, operative notes may be used not only as a reminder of intraoperative findings, but also as useful information to guide future treatment and communication with other physicians. The checklist may lead to appropriate use of immediate intravesical chemotherapy for the right patients and decrease intravesical recurrences. A previous study reported that operative reports lacking specific important elements were associated with higher rates of major complications than those with more complete reporting [10]. Routine operative documentation, including the fundamental goals of surgery, may increase the potential for complication-free surgery.

There were several limitations in the present study. This was a retrospective analysis with a small cohort and non-randomized study design. Many surgeons performed TURBT; however, the attending surgeons were always supervised by a senior staff member and other residents. The follow-up periods of checklist group were shorter compared to that of the non-checklist group. Nevertheless, the results obtained suggest that the introduction of a checklist into clinical practice increases the quality of operative reports and facilitates complete and accurate TURBT, resulting in lower disease recurrence rates.

## Conclusions

The introduction of a checklist is recommended for the standardization of TURBT and increases in the quality of operative reporting, and it may also improve oncological outcomes.

## Supporting information

S1 Fig Kaplan–Meier curves of the intravesical recurrence-free survival rates for TURBT using with and without surgical checklist groups in (A) the patients received intravesical therapy and in (B) the patients not received intravesical therapy.
(TIF)

S1 Table Checklist for TURBT. (DOCX)

S2 Table Multivariate analysis with Cox proportional hazards modeling for disease progression. (DOCX)
