# Supplementary figures and images for: Quality improvement in managing patients with non-muscle-invasive bladder cancer by introducing a surgical checklist for transurethral resection of bladder tumor
Source: PLoS One. 2022 Oct 27;17(10):e0276816. doi: 10.1371/journal.pone.0276816 (PMC9612454; doi:10.1371/journal.pone.0276816)

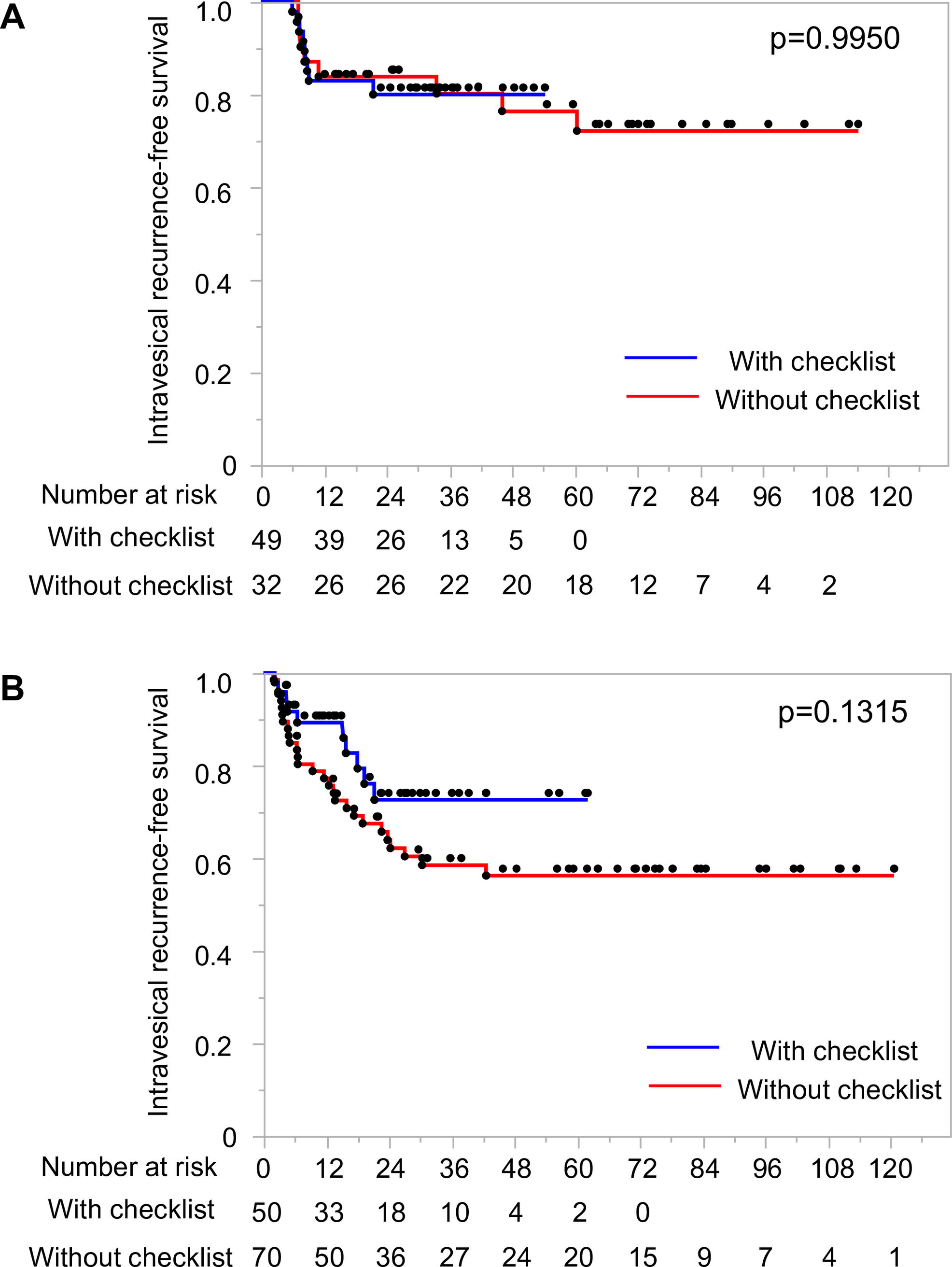

Supplement: S1 Fig — Kaplan–Meier curves of the intravesical recurrence-free survival rates for TURBT using with and without surgical checklist groups in (A) the patients received intravesical therapy and in (B) the patients not received intravesical therapy. (TIF) [file pone.0276816.s001.tif]
