# Supplementary material for: Quality improvement in managing patients with non-muscle-invasive bladder cancer by introducing a surgical checklist for transurethral resection of bladder tumor
Source: PLoS One. 2022 Oct 27;17(10):e0276816. doi: 10.1371/journal.pone.0276816 (PMC9612454; doi:10.1371/journal.pone.0276816)
Supplement: S1 Table — (DOCX) [file pone.0276816.s002.docx]

Supplementary Table 1

| **Checklist for TURBT** |  |
| --- | --- |
| 1. The purpose of this procedure | Treatment for primary tumor・Treatment for recurrent tumor・Second TUR・Protocol biopsy after BCG treatment・Others ( ) |
| 2. Tumor number | 0・1・2-7・≥8・Uncountable・Others ( ) |
| 3. Largest tumor diameter | <3cm・≥3 cm・Unmeasurable・Others ( ) |
| 4. Macroscopic tumor appearance | Papillary pedunculated・Papillary broad-base・Nodular pedunculated・Nodular broad-base・Flat・Ulcer |
| 5. Assess for presence of carcinoma in situ | Yes・No |
| 6. Random biopsy of the sites without abnormal findings (Select the biopsy locations) | Yes (Anterior・Posterior・Right lateral side・Left lateral side・Dome・Trigone)・No |
| 7. Biopsy of the prostatic urethra | Yes・No |
| 8. Clinical T stage | cT0・cTis・cTa-1・cT2・cT3・cT4 |
| 9. Bimanual exam under anesthesia | Yes・No |
| 10. Visually complete resection | Yes・No |
| 11. Visualization of detrusor muscle in resection base | Yes・No |
| 12. Visual evaluation for perforation | Yes・Near perforation・No |
