# Supplementary material for: Quality improvement in managing patients with non-muscle-invasive bladder cancer by introducing a surgical checklist for transurethral resection of bladder tumor
Source: PLoS One. 2022 Oct 27;17(10):e0276816. doi: 10.1371/journal.pone.0276816 (PMC9612454; doi:10.1371/journal.pone.0276816)
Supplement: S2 Table — (DOCX) [file pone.0276816.s003.docx]

Supplementary table 2

| **Variables** | **N** | **Multivariate** | |
| --- | --- | --- | --- |
|  |  | **Hazard ratio (95% CI)** | ***P*-value** |
| Tumor status  primary  recurrent | 173  28 | Ref  0.4237 (0.0468－3.8464) | 0.4455 |
| Number of tumors  single  multiple | 86  114 | Ref  5.5325 (0.6471－47.302) | 0.1182 |
| Tumor size  <3 cm  ≥3 cm | 167  31 | Ref  0.6302 (0.0771－5.1501) | 0.6667 |
| Histology  UC  UC with variant histology | 190  11 | Ref  Incalculable | 0.9992 |
| TUR with checklist  no  yes | 102  99 | Ref  0.8236 (0.1935－3.5017) | 0.7923 |
| Second TUR  no  yes | 120  81 | Ref  0.4337 (0.0905－2.0794) | 0.2962 |
| Adjuant therapy (BCG  or chemotherapy)  no  yes | 120  81 | Ref  3.8934 (0.7183－21.104) | 0.1150 |

BCG = bacillus Calmette-Guérin; CIS = carcinoma in situ; TUR = transurethral resection; UC = urothelial carcinoma.
